# The complete chloroplast genome of *Camellia semiserrata* Chi. (Theaceae), an excellent woody edible oil and landscaping species in South China

**DOI:** 10.1080/23802359.2021.1976690

**Published:** 2021-09-22

**Authors:** Le Dong, Xin Yin, Bin Huang, Tian Li, Jian Jian Huang, Qiang Wen

**Affiliations:** a Jiangxi Provincial Key Laboratory of Camellia Germplasm Conservation and Utilization, Jiangxi Academy of Forestry, Nanchang, China; b Co-Innovation Center for Sustainable Forestry in Southern China, Nanjing Forestry University, Nanjing, China

**Keywords:** *Camellia semiserrata*, chloroplast genome, phylogenetic analysis

## Abstract

*Camellia semiserrata* is a woody plant that produces excellent edible oil and is a common landscaping species in South China. The complete chloroplast genome of *C. semiserrata* was sequenced, assembled, annotated, and characterized using the Illumina MiSeq platform in this study. The chloroplast genome is 156,968 bp (37.32% GC) and contains a large single copy (LSC) region (86,634 bp), a small single copy (SSC) region (18,272 bp), and a pair of inverted repeat (IR) regions (26,031 bp). It encodes a total of 117 genes, including 81 protein-coding genes, four ribosomal RNA genes, and 32 transfer RNA genes. The phylogenetic tree fully resolved *C. semiserrata* in a clade with *C. reticulata*, *C. mairei*, and *C. pitardii*. This study contributes to bioinformatics and further phylogeny and conservation studies as well as provides a theoretical basis for the molecular identification of *C. semiserrata*.

*Camellia semiserrata* Chi. is mainly distributed in the south of China (Wu et al. 2008), belonging to sect. *Camellia* in the genus *Camellia* L. (Theaceae) (Ding et al. 2012). Due to a long flowering period and the good quality of seed oil, *C. semiserrata* has both ornamental and economic values and shows great market potential (Rong et al. 2021). At present, the phylogenetic history among sect*. Camellia* species has not yet been fully resolved (Chang and Ren 1998; Ming 1998). As the chloroplast genome is more conservative than the nuclear genome and mitochondrial genome in terms of genome size, genome structure, and gene content (Palmer 1985), the genes obtained from the chloroplast genome have been used to distinguish species and for phylogenomic analysis (Gu et al. 2019). Here, we analyzed the chloroplast genome of *C. semiserrata* to determine its evolutionary systematics and to document the genetic history of the germplasm resources of *C. semiserrata*.

Young and fresh leaves of *C. semiserrata* were collected in the National Gene Bank of the *Camellia* Germplasm Resource (Jiangxi, China; coordinates: 28°44′21.26″N, 115°49′5.42″E), and the specimens were deposited in the Key Laboratory of *Camellia* Germplasm Conservation and Utilization, Jiangxi Academy of Forestry (contact person: Dong le, nxqtxdl@163.com; specimen voucher: JX20210101). The chloroplast DNA Isolation Kit (Sigma, St. Louis, MO) was used to extract the chloroplast genomic DNA of *C. semiserrata* following the manufacturer’s instructions. The DNA was sequenced with the Illumina MiSeq using 250 bp paired-end sequencing, then raw data were trimmed with Trimmomatic 0.39 (Bolger et al. 2014). Finally, about 1.31 Gb clean data (SRR15294181) were assembled into the complete chloroplast genome with GetOrganelle v1.7.4 pipeline (Jin et al. 2020) using the reference genome of *C. chekiangoleosa* (GenBank accession number MG431968). The genome annotation was performed with GeSeq (Tillich et al. 2017), and manually corrected through aligning to the genome annotations of *C. chekiangoleosa* using Geneious v9.0.2 (http://www.geneious.com). The chloroplast genome of *C. semiserrata* is deposited in the NCBI database under the accession number MZ403753.

The complete chloroplast genome of *C. semiserrata* is a circular molecule of 156,968 bp in length, with a large single copy (LSC) region of 86,634 bp, a small single copy (SSC) region of 18,272 bp, and a pair of inverted repeat (IR) regions of 26,031 bp. The total GC content of the chloroplast genome is 37.32%, and the corresponding values of LSC, SSC, and IRs are 35.33%, 30.56%, and 42.99%, respectively. The chloroplast genome encodes 117 unique genes, including 81 protein-coding, 32 tRNA, and four rRNA genes. There are nine protein-coding, four rRNA, and seven tRNA genes repeated in the IR region. In addition, comparative analysis revealed that the chloroplast genome structure and gene locations of *C. semiserrata* are similar to other related chloroplast genomes of *Camellia*, such as *C. azalea* (GenBank accession number KY856741) and *C. ptilophylla* (GenBank accession number MG797642).

To study evolutionary relationships, the phylogenetic tree was constructed based on chloroplast genome sequences of *C. semiserrata* and other 26 species of *Camellia* downloaded from NCBI GenBank using the GTR + I+G substitution model in MrBayes v3.2.6 (Fredrik et al. 2012), and designating *Symplocos ovatilobata* and *Symplocos costaricana* as the outgroup. The result showed that *C. semiserrata*, *C. reticulata*, *C. mairei*, and *C. pitardii* belong to the subsect. *Reticulata* clustered together, while *C. japonica* and *C. chekiangoleosa* belong to the subsect. *Lucidissima* clustered with another branch (Figure 1). It showed that there are obvious differences between subsect. *Reticulata* and subsect. *Lucidissima* in sect. *Camellia*, which indicated that our results accord with the Flora Reipublicae Popularis Sinicae on the classification of subsections in sect. *Camellia* (Chang and Ren 1998). Besides, we also found that sect. *Camellia* was closely related to sect. *Oleifera* and sect. *Paracamellia*, which also reflected the flower color cannot be used as the basis for the classification of sect. *Camellia* (Ming 2000). This study provides a reference for the phylogenetic relationship of *C. semiserrata* and sect. *Camellia* species.

**Figure 1.**
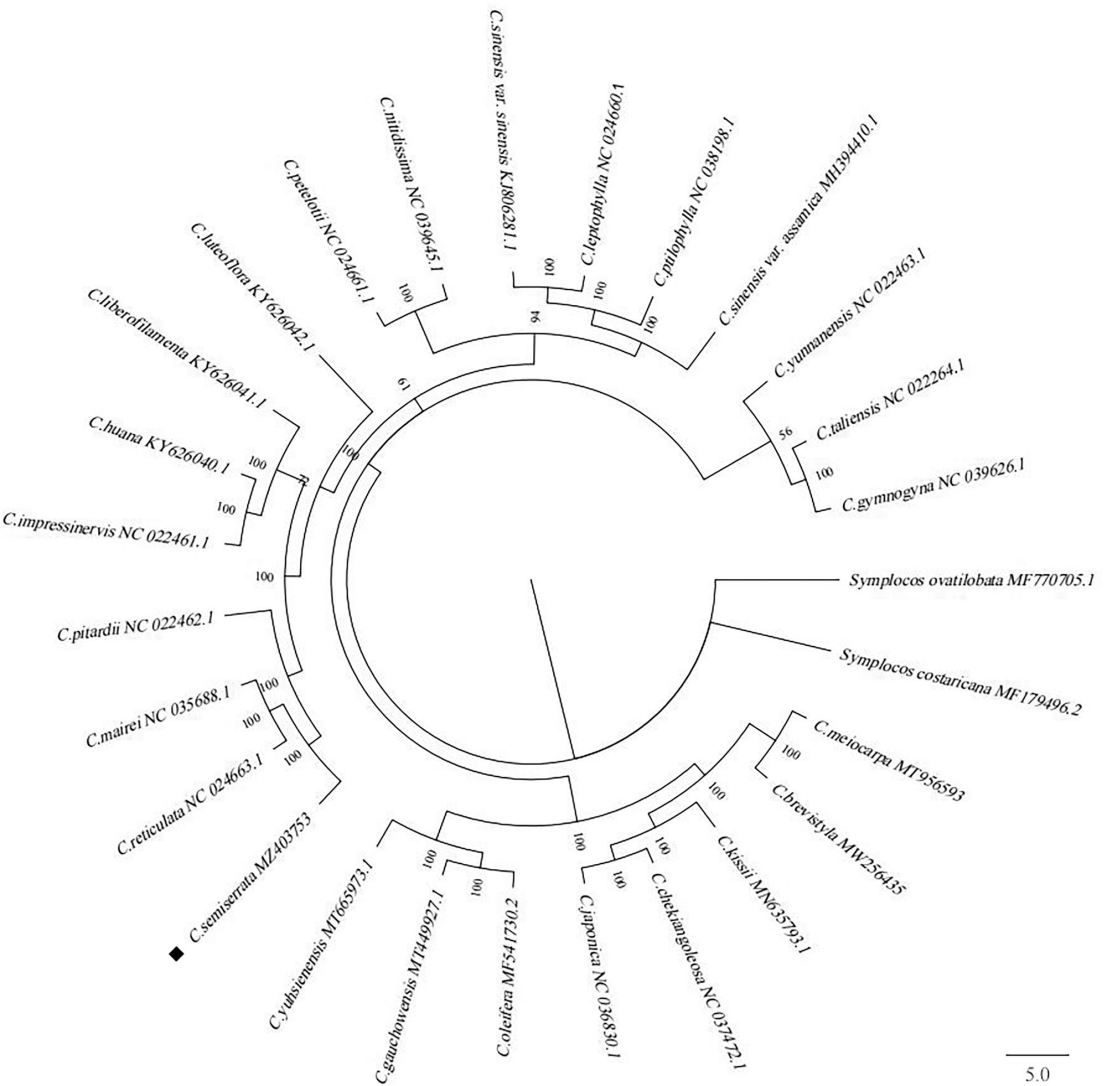
The Bayesian inference (BI) phylogenetic tree constructed based on the complete chloroplast genome sequences of 27 *Camellia* species. Number near the nodes represents the posterior probabilities.

## Data Availability

The genome sequence data that support the findings of this study are openly available in GenBank of NCBI at https://www.ncbi.nlm.nih.gov/ under the GenBank accession no. MZ403753. The associated BioProject, SRA, and BioSample numbers are PRJNA750633, SRR15294181, and SAMN20475199, respectively.
